# Report of Two Cases of Typhoid Fever

**Published:** 1899-10

**Authors:** J. M. McClanahan

**Affiliations:** Retreat, S. C.


### REPORT OF TWO CASES OF TYPHOID FEVER.

By J. M. McCLANAHAN, M.D.,

Retreat, S. C.

June 14, 1899, 10 A. M. G. F. M., male, white, aged twenty-five years, employed at saw-mill; was brought to my office and gave following history of illness: Eight days ago "commenced feeling badly," unfit for work but not sick enough to go to bed; had frontal headache a week, bowels loose, temperature 102 degrees, etc. Ordered him home and to bed and gave him two grains calomel and one grain salicylic acid every three hours, with opium powders to check bowels if actions were more than three daily; called next morning and found conditions unchanged. Temperature 102 degrees; tongue slightly coated; bowels acting two to three times daily; actions like "yellow of egg"; some tenderness and resonance in right iliac region. Same treatment continued with addition of ten m. aromatic sulphuric acid three times daily, and quinia sulphate grs. vi., to be taken every four hours should the fever increase. His temperature never exceeded 102 degrees. I visited him every third or fourth day. Only change made in treatment was to reduce frequency of calomel and salicylic acid doses to three times daily after fourth or fifth day of treatment. He was dismissed and all medicine discontinued on the 30th. Two weeks after his discharge he resumed his duties as wagoner at saw-mill.

Mrs. K., white, aged forty-six, was indisposed a week before she took to her bed. Saw her on August 31st, 3 P. M.; frontal headache very severe; tongue coated, bowels loose, "yellow of egg" discharges; resonance decided and tenderness in right iliac region. Temperature 104 degrees. Prescribed 10 grs. acetanilid to relieve or mitigate headache, to be repeated in two hours; calomel 2 grs., salicylic acid 1 grain, every three hours; left opium to check bowels if calomel purged excessively. September 2, 8 A. M., temperature 104; head still aching, but not severely; other symptoms same; treatment continued. There was no change in her treatment except ordered lemonade. On September 3d, 2 P. M., temperature 102 degrees. On 5th, 8 A. M., 102 degrees. On 8th, 2 P. M., 101 degrees. On the 14th, normal; tongue clean; sleeps well; bowels have never acted more than four times in twenty-four hours. Only used three or four doses of opium. Discharged.

Both above patients were supposed to have imbibed the germs of the disease from same well; both were typical cases; both ran a course of about two weeks from time of taking to bed. My objection to Woodbridge treatment has been the excessive frequency of doses. If further observations sustain the conclusions to which above treatment points—*i. e.*, that the calomel and salicylic acid destroy germs in small intestines, the objection of such frequent doses is eliminated. The difficult solubility of the mercury and salicylic acid may permit them to pass through the stomach and reach the seat of ulceration in ileum. I have watched closely and in only one case discovered the slightest evidence of ptyalism and that after the convalescence of patient.

Will salicylic acid administered with mercury prevent salivation, or was it prevented by the frequent action of bowels that always accompanies typhoid fever? I am now treating a case where I desire the alterative effect of mercury, with protoiodide mercury $\frac{1}{4}$ grain, salicylic acid 1 grain, three times daily.

